# Mind–Body Interventions for Pediatric Inflammatory Bowel Disease

**DOI:** 10.3390/children4040022

**Published:** 2017-04-03

**Authors:** Ann Ming Yeh, Anava Wren, Brenda Golianu

**Affiliations:** 1 Department of Pediatrics, Division of Pediatric Gastroenterology, Hepatology and Nutrition, Stanford University, Palo Alto, CA 94304, USA; awren2@stanford.edu; 2 Department of Anesthesia, Perioperative and Pain Medicine, Stanford University, Palo Alto, CA 94304, USA; bgolianu@stanford.edu

**Keywords:** mind-body, inflammatory bowel disease, pediatric, biofeedback, psychotherapy, hypnosis, yoga, relaxation

## Abstract

Pediatric inflammatory bowel disease is an autoimmune disease that causes chronic inflammation of the gastrointestinal mucosa. There is emerging evidence that the brain–gut connection affects inflammatory bowel disease (IBD) patients more than previously thought. This is evidenced by comorbid mood disorders, irritable bowel symptoms concurrent with quiescent IBD, and the potential of psychosocial stressors to trigger IBD flares. Mind–body interventions such as psychotherapy, relaxation, mindfulness, biofeedback, yoga, and clinical hypnosis offer an adjunct to standard medical treatment for IBD. We will review the current evidence base for these mind–body interventions in the treatment of pediatric IBD, illustrate a case study, and offer suggestions for future research for this promising field.

## 1. Introduction

Inflammatory bowel disease (IBD) is a chronic and relapsing inflammatory condition involving all or part of the gastrointestinal (GI) tract. The most common forms of IBD are Crohn’s Disease (CD) and Ulcerative Colitis (UC), where CD may involve the entire GI tract and UC involves only the large intestine. The pathophysiology of IBD is thought to be multifactorial, involving a complex interplay between genetic predisposition, the gut microbiota, gut mucosal integrity, the immune system, and environmental triggers such as nutrition and psychosocial stressors [1,2]. Conventional treatments for pediatric IBD include anti-inflammatory pharmacologic therapies (5-aminosalicylic acid derivatives), corticosteroids, immunomodulators (6-mercaptopurine, methotrexate), and biologics [3]. Nutritional therapies such as enteral nutrition can also be effective for small bowel Crohn’s disease [4,5,6].

Patients with IBD commonly seek integrative and complementary medicine treatment due to beliefs that medications are not effective, concern about medication side effects/risks, and interest in adjunct therapies to optimize disease management and help to improve symptoms and quality of life [7]. Integrative medicine is considered a healing-oriented medicine that reaffirms the importance of the relationship between practitioner and patient, focuses on the whole person, and utilizes empirically supported therapeutic approaches. According to the National Institute of Health, integrative medicine is an umbrella term that can include various modalities such as natural products (e.g., herbs and supplements), whole systems medicine (e.g., traditional Chinese medicine, Ayurveda), and mind–body interventions (e.g., relaxation, yoga, clinical hypnosis). The term integrative refers to the coordinated approach of utilizing conventional and complementary therapies together in an integrative way [8].

Over the past decade, mind–body interventions (MBI) have received increased attention. These interventions are categorized as those that ‘employ a variety of techniques designed to facilitate the mind’s capacity to affect bodily function and symptoms’ [9]. Mind–body interventions are thought to be dynamic, and some definitions of MBI include manipulative modalities such as acupuncture, massage, and spinal manipulation. For the purposes of this review, however, we will focus on non-manipulative MBI, including psychotherapy, relaxation techniques, mindfulness-based therapies, biofeedback, yoga, and clinical hypnosis. Mind–body interventions are emerging as popular treatment modalities amongst IBD patients [10] and provide safe, inexpensive, non-pharmacologic ways to target the autonomic nervous system by decreasing the stress response and increasing the relaxation response.

The aim of this paper is to: (1) provide background information and a rationale for the use of mind–body interventions in pediatric IBD populations; (2) provide a brief overview of key mind–body interventions for pediatric IBD; (3) review the safety and efficacy of these mind–body interventions and highlight areas for future research; and (4) present a sample case demonstrating a multidisciplinary approach to mind–body IBD treatment.

Regarding the criteria for considering studies for this review, all controlled clinical trials, quasi-randomized trials, and non-randomized trials including pediatric patients with UC or CD and mind–body interventions were included. The search criteria were expanded to include adult patients with IBD or youth with irritable bowel syndrome (IBS) when no literature was available in pediatric IBD samples. Studies published between 1980 and 2017 were considered in this review. Pubmed was utilized as the primary search engine. The search strategies included terms related to pediatrics, IBD, IBS, mind–body interventions identified for this review, and common synonyms for the aforementioned terms.

## 2. Background

While the pathophysiology of IBD is thought to be due to interrelated factors, increasing evidence has shown the intimate connection between the brain and the gut and the effect of this bidirectional interaction on gut and psychological health [11]. It is noteworthy to highlight that IBD patients readily identify stress as a contributing factor to disease flares [12,13]. Numerous studies have noted a significant association between perceived stress, negative mood, and major life events and disease relapse, hospitalization, and abdominal surgery risk [12,14]. Prospective studies show that exposure to stressful events as well as perceived stress (i.e., the individual’s view of a demand relative to their resources) may contribute to relapse risk [15,16,17]. One study found that pediatric patients with IBD who had low stress levels and used non-avoidant coping strategies had fewer relapses [15], suggesting the potential benefit of stress management interventions in this population.

The bidirectional brain-gut relationship is especially notable in youth who have IBD with associated IBS-type symptoms. Despite adequate control of their IBD, patients may continue to suffer from refractory IBS symptoms. At times, this situation may present a diagnostic and treatment challenge, as it may be difficult to distinguish between ‘true’ IBS symptoms versus a subclinical form of IBD. Children with IBD in clinical remission, as defined in the abbreviated-PCDAI (pediatric Crohn’s disease activity index) or the PUCAI (pediatric ulcerative colitis activity index), have a 6.4% prevalence of IBS-type symptoms. In patients who have biochemical markers of quiescent or minimal disease, with a calprotectin of <250 μg/g, IBS symptom prevalence is 16.1% (95% confidence interval (CI): 7.6–25.8%; Crohn’s disease: 16.7%; ulcerative colitis: 10.8% [18]. A meta-analysis of 1703 patients showed that patients with IBD had a higher prevalence of IBS symptoms, compared to non-IBD controls [19].

Patients in IBD remission may suffer from a variety of somatic complaints, including recurrent abdominal pain, urgency, diarrhea, constipation, bloating, gas, and cramping sensations. Symptoms of pain and discomfort may persist even after inflammation is adequately controlled due to visceral hyperalgesia, somatic-parietal abdominal pain, referred pain, strictures or adhesions, small intestinal bacterial overgrowth (SIBO), dysmotility, and central sensitization [20]. Factors that may increase central sensitization include disordered sleep, social stressors, and the presence of anxiety or depression [20]. IBD and IBS have overlapping and interacting mechanisms of illness. Subsets of IBS patients may have low grade inflammation, and some studies suggest that IBS and IBD exist on a similar disease continuum [21,22]. Furthermore, both IBD and IBS patients may have derangements in the gut microbiota that lead to disease [23,24,25].

A multidisciplinary approach to the management of IBD and IBS symptoms is essential to successful symptom improvement and disease treatment [26,27,28]. This approach includes thorough assessment and management by a pediatrician in consultation with a gastroenterology specialist to ensure adequate control of inflammation and gut physiological factors. Additional providers include a dietician to assess nutritional concerns; a pain specialist if pain is a presenting complaint; a physical or occupational therapist if there is significant deconditioning or loss of quality of life; and a clinical psychologist, pediatric pain psychologist, or other mental health therapist if there are psychosocial, adjustment, or symptom management concerns. An effective multidisciplinary team will work in tandem to guide appropriate interventions and disease modifying therapies for patients, optimize their physiologic and psychological wellbeing, and help to improve and sustain their ongoing quality of life [29].

IBD patients, specifically those with persistent IBS symptoms despite quiescent inflammation, may benefit from integrating mind–body interventions into multidisciplinary care. Research has demonstrated that mind–body interventions are commonly utilized by patients with IBS and IBD and may help to decrease anxiety and improve coping abilities [10]. Of note, up to 84% of pediatric patients with IBD already utilize some sort of integrative medicine therapy, according to a single-center survey at a tertiary care center [10]. Among these, 61% of patients used stress management and 35% utilized special exercises (e.g., yoga, swimming). In another survey of 67 adolescents with IBD, 40% used relaxation and 21% used guided imagery once a day to once a week for symptom management [30]. Younger adolescents were more likely to regularly use meditation, and adolescents with more severe disease were more willing to consider using relaxation as an adjunct therapy. Given the high utilization of mind–body interventions among pediatric IBD patients and the potential efficacy of MBI for this population, providers should be knowledgeable and open to discussing common mind–body interventions.

## 3. Mind–Body Interventions

### 3.1. Psychotherapy

Patients with IBD endorse elevated levels of emotional distress and poor quality of life compared to the general population [28,31]. Anxiety and depressive disorders have been documented to be higher among patients with IBD compared to non-IBD cohorts [31,32,33]. In pediatric IBD populations, high rates of anxiety and depression have been documented and there is an increased risk of developing depression compared to youth with other chronic illnesses and healthy controls [33,34,35]. Symptoms of anxiety and depression have been negatively associated with psychological well-being and disease-related outcomes in IBD populations, including lower quality of life, increased perceived stress, IBD relapse, inflammation, pain, poor medication adherence, and increased risk of surgery [28,33,36,37,38,39]. Unfortunately, psychiatric comorbidities are often under-diagnosed and undertreated across the lifespan. Recently, there has been growing support for multidisciplinary IBD treatment in order to treat the whole patient, including psychological interventions for youth and adults with IBD [28]. Psychological interventions have received increasing attention in the mind–body IBD literature [7], given the comorbidity between psychological conditions and IBD and the preliminary efficacy of such treatments on both psychological and physical outcomes.

Psychological interventions aimed at improving emotional distress and disease outcomes among IBD patients have generally focused on psychotherapy, stress management, hypnosis, and psychoeducation [40]. Over the past decade, numerous systematic reviews and meta-analyses have explored the effect of psychological interventions on children, adolescents, and adults with IBD. Overall, the findings have been mixed. A Cochrane review from 2011 [41] concluded that there was no evidence for psychological interventions in adults with IBD; however, the authors noted more empirical evidence for the use of psychotherapy among pediatric populations with IBD. Specifically, such interventions were found to improve depression and quality of life among youth [41]. More recent reviews have found psychological interventions to have some benefit on psychological outcomes and growing potential to reduce physical symptoms such as pain and fatigue, relapse rate, hospitalization, and medication adherence [40,42]. These mixed findings have been partially attributed to the limited body of research, comparing interventions with varying psychological content and methodological limitations.

#### Cognitive Behavioral Therapy

Cognitive behavioral therapy (CBT) is one type of psychological intervention that has demonstrated particular promise among pediatric populations with IBD. While there are many types of psychotherapy, CBT is the most common and has the greatest evidence base in pediatric IBD populations [35,43]. CBT is based on the premise that emotions, thoughts, and behaviors are interconnected and that psychological functioning can improve by working to understand and change maladaptive thoughts and behaviors. Significant literature supports the use of CBT among children with anxiety and mood disorders [44,45], and growing research has demonstrated the efficacy of CBT protocols among youth with chronic illnesses [46].

Szigethy and colleagues conducted some of the first studies exploring the effectiveness of CBT among youth with IBD [47]. They modified a manual-based CBT protocol to address the impact of physical illness on youth with depression. The intervention focused on physical illness narrative, social skills training, and family education. The protocol consists of nine individual modules focused on behavioral activation, cognitive restructuring, and problem-solving skills and three family sessions focused on psychoeducation and family problem-solving skills. An early pilot study demonstrated promising results among 11 adolescents (12–17 years). Specifically, there were significant reductions in child and parent-reported depression symptoms pre- to post-treatment (*p* < 0.001; 0.65–0.92 effect size), as well as significant improvements in global adjustment (*p* < 0.01) and physical functioning (*p* < 0.05) among youth [47]. Despite these positive results, there were several study limitations, including the small sample size and the lack of a control group and long-term follow-up, limiting assumptions of causality and generalizability.

Later larger randomized controlled groups by Szigethy et al. [48] demonstrated that, among 41 youth with IBD, the protocol could lead to clinically significant improvements in depression symptoms (*p* < 0.01; 1.01 effect size) and functioning (*p* < 0.05, 0.86 effect size), compared to treatment as usual. Of note, these benefits persisted one-year post-treatment. When the protocol was compared to supportive non-directive therapy (SNDT), the results showed that both interventions equally reduced depression symptoms (37.3% for CBT; 31.9% for SNDT) among 217 youth [43]. There were also significant improvements across groups on measures of global functioning, quality of life, and disease activity; however, the CBT intervention showed a significantly greater reduction in IBD activity compared to SNDT (*p* = 0.04). Disease activity was measured by the PCDAI and PUCAI disease activity scoring scales for CD and UC, respectively. Lastly, follow-up analyses on a smaller study sample of youth with CD (*N* = 70) showed that both CBT and SNDT were associated with decreased GI-related health care utilization, specifically GI-hospitalization frequency, inpatient days, ER visits, and endoscopies [49]. Overall, these results support CBT as a beneficial adjunct to the medical management of IBD among pediatric patients.

Recent research supports Szigethy and colleagues’ findings over the past decade, demonstrating the benefits of CBT protocols for youth with IBD and emotional distress. Of note, Levy et al. [50] randomized 187 youth (8–17 years) to a brief three session CBT intervention or an education support condition and conducted follow-up assessments up to one year post-treatment. The findings demonstrated that the CBT intervention led to significantly more pre- to post-treatment improvement compared to the control group on measures of pain coping abilities (*p* < 0.05), IBD-related quality of life (*p* < 0.05), parent-reported solicitous behavior (*p* < 0.05), and missed days of school in the six months following treatment (*p* < 0.05). Children reported significant improvement in depression symptoms post-treatment with no significant between group differences (*p* = 0.10). Interestingly, exploratory sub-group analyses demonstrated that children in the CBT intervention with higher levels of flares pre-baseline had significantly fewer flares post-treatment compared to the control (16.7% versus 52.9%; *p* < 0.05). Future studies should further explore the possibility that brief CBT protocols can affect disease activity in a larger sample of youth with IBD using more rigorous methodology, such as PCDAI/PUCAI measures of disease activity, and the concurrent measurement of calprotectin and inflammatory cytokines. The strengths of this study include the large sample size, controlling for time and attention in the educational support condition, and developing a brief intervention to address the accessibility and adherence issues common to psychotherapy protocols.

Currently, a multicenter randomized controlled trial (RCT) is underway to investigate the efficacy of a CBT protocol in reducing symptoms of depression and anxiety and improving quality of life and disease course in youth with IBD [51]. Larger RCTs such as this are greatly needed in order to address past study limitations and further assess the feasibility, efficacy, and generalizability of CBT interventions for youth with IBD and co-occurring emotional distress.

Overall, there is promising preliminary evidence for the feasibility of CBT interventions in tertiary pediatric GI/IBD clinics and the efficacy of such protocols in improving psychological wellbeing and disease-related outcomes. Effective non-pharmacological treatments for emotional distress are especially important given the comorbidity between IBD and depression and anxiety and the potential for antidepressants to exacerbate GI symptoms and complicate medical regimens [47].

### 3.2. Relaxation and Stress Management

Given the relationship between stress, emotional distress, and negative IBD-related outcomes, researchers have explored the efficacy of relaxation and stress-management protocols for IBD patients. The goal of such interventions is to teach patients skills to relax and reduce daily stress in order to support improved psychological and physical wellbeing. The content of these interventions varies, as well as the format (individual versus group sessions), delivery (nurse, psychiatrist, psychologist), and duration (one session versus eight weekly sessions). Relaxation-based interventions usually focus on teaching patients a subset of relaxation strategies, such diaphragmatic breathing techniques, guided imagery (e.g., focus on imagination and distraction), progressive muscle relaxation (PMR) (e.g., focus on building and releasing muscle tension), and autogenic training (i.e., focus on physical manifestations of relaxation in the body like heaviness in the musculoskeletal system or warmth of the circulatory system) [41]. Stress management protocols tend to include psychoeducation (e.g., related to IBD and treatment; stress and relaxation response) and a variety of relaxation and coping skills (e.g., problem solving, communication, identifying and modifying negative thoughts). Exercise and diet support are also integrated into some stress management treatments [40,41].

The majority of research investigating the use of relaxation and stress management interventions in IBD populations has been conducted in adult samples. Randomized controlled trials investigating the efficacy of PMR and guided imagery-based relaxation protocols have shown significant improvements in psychological functioning, quality of life, and pain variables among adults with IBD [52,53]. Stress management interventions have shown mixed efficacy. Some RCTs have demonstrated that stress management protocols can improve psychological functioning [54,55,56] and disease-related outcomes [55,57,58], while other studies show no significant changes in IBD outcomes across conditions [59].

Research exploring more integrative CBT interventions (CBT with greater emphasis on relaxation-based coping skills) has shown promise in pediatric patients with IBD. McCormick and colleagues [60] developed a one-day CBT intervention for adolescents and parents focused on disease-related coping skills, pain management, relaxation, communication, and limit setting (*N* = 24 parent–child dyads). Treatment was followed by six weeks of web-based skill review that focused on weekly homework, coping skills, and group chat sessions related to treatment modules. Separate adolescent and parent sites were created. Following the integrative CBT treatment, there were significant improvements in child and parent-reports of somatic symptoms (*p* < 0.01) and adaptive coping (*p* < 0.05). While there were no significant differences between the CBT and wait-list control condition, no changes in the above symptoms were found for the control group. Despite preliminary support for the intervention among adolescents with IBD and their parents, there was a lack of random assignment to groups, limiting conclusions about intervention efficacy. Overall, it appears that no studies to date have explored the efficacy of relaxation or stress management interventions in pediatric IBD populations.

Future research exploring the feasibility and efficacy of relaxation-focused interventions among pediatric samples is needed. Brief relaxation protocols could be particularly helpful in pediatric GI clinics, as they could be easily delivered by a variety of providers and integrated into multidisciplinary care.

### 3.3. Mindfulness

Over the past decade, mindfulness-based interventions have received increasing attention in mind–body research and are emerging as a point of interest among researchers exploring psychotherapeutic interventions for patients with IBD. Mindfulness-based stress reduction (MBSR) and mindfulness-based cognitive therapy (MBCT) are typically the most common mindfulness treatments in clinical and research settings. Across these protocols, the primary treatment aim is to teach patients mindfulness, which is the ability to be aware of the present moment and one’s thoughts, emotions, and body sensations in a purposeful, non-judgmental manner [61,62]. Mindfulness is usually practiced via a series of breathing, meditation, and movement exercises, all of which support non-judgmental and compassionate awareness. Bringing one’s focus and attention to the present can buffer one from ruminating on negative cognitions (e.g., catastrophizing about IBD flares) or bothersome physical sensations (e.g., abdominal pain).

To date, it appears that mindfulness-based interventions for patients with IBD have been exclusively conducted in adult samples. One of the first randomized controlled studies investigated the feasibility and efficacy of an eight week mindfulness-based stress reduction (MBSR) group among 55 patients with ulcerative colitis compared to an attention control condition with mind–body psychoeducation [63]. MBSR was developed by John Kabat-Zinn and consists of eight weekly 2.5 h sessions, weekly homework, and a seven hour weekend session, all focused on promoting mindfulness in daily life. Results demonstrated that the MBSR intervention was highly feasible and acceptable among IBD patients; however, the protocol did not impact psychological or disease outcomes compared to the control condition. Interestingly, among patients who flared during the study, those randomized to the MBSR condition reported significantly higher quality of life compared to the control condition (*p* = 0.001). Additionally, post-hoc analyses demonstrated that MBSR may be effective in reducing flares among patients with heightened states of stress, supporting the approach of tailoring treatments to patient characteristics as is routinely done with pharmacotherapy [63].

A more recent controlled trial [64] provided preliminary support for the feasibility, acceptability, and efficacy of an eight week modified MBSR protocol for adults with IBD (*N* = 60). Post-treatment, there were significant improvements in anxiety (*p* < 0.05), depression (*p* < 0.05), quality of life (*p* < 0.01), and mindfulness (*p* < 0.01), compared to a treatment as usual control condition. Of note, significant reductions in depression and improvements in mindfulness were present six months following the intervention (*p* < 0.001). The corresponding effect sizes ranged from medium (*d* = 0.56) to large (*d* = 1.27), providing support for clinically meaningful outcomes. However, additional randomized controlled trials are needed to further support these findings.

Schoultz and colleagues [65] have also explored the efficacy of a MBCT intervention among 44 adults with IBD. MBCT was developed by Zindel Segal and colleagues and is largely based on MBSR [66]. It is an eight-week group treatment that integrates mindfulness and cognitive therapy techniques to support stress management and self-care. Schoultz et al. [65] found that MBCT led to significant improvements in depression (*p* < 0.05), trait anxiety (*p* < 0.05), and dispositional mindfulness (*p* < 0.05) among patients with IBD, compared to a wait-list control condition. These findings persisted at a six-month follow-up assessment. No changes were noted on disease activity and IBD quality of life. Thomas and colleagues also developed a form of psychotherapy, entitled multi-convergent therapy (MCT), that combines mindfulness with cognitive and behavioral techniques [67]. A randomized controlled pilot study assessed the efficacy of MCT among 66 adults with IBD. The results demonstrated significant pre- to post-treatment improvements in the MCT condition, compared to the control on quality of life among IBD patients with IBS-type symptoms (determined by Rome III criteria at baseline) (*p* = 0.021) [68]. These findings offer further support to the notion that mindfulness protocols could be effective for sub-groups of IBD patients.

While, to our knowledge, there is no published research investigating the use of mindfulness-based interventions among youth with IBD, preliminary support for mindfulness protocols in adult IBD populations and youth with IBS [69] suggest that this could be a promising line of future research. Going forward, investigators should explore the feasibility and efficacy of age appropriate mindfulness treatments among children and adolescents with IBD, particularly in patients with heighted states of stress, concurrent psychological diagnoses, or IBS-IBD symptoms. This research should also address some of the methodological limitations highlighted in the adult literature. Specifically, future studies should include a bigger sample size and multiple control groups and include attention and wait list control, assess treatment adherence and engagement, and collect psychological, physiological, and disease activity-related outcomes.

### 3.4. Biofeedback

Biofeedback is a process in which the electronic monitoring of normally automatic bodily functions (e.g., heart rate, blood pressure, skin temperature) is used to train a patient to acquire voluntary control of that function. The autonomic nervous system is the primary target of biofeedback, particularly the sympathetic nervous system, or the body’s ‘fight or flight’ system. The autonomic nervous system received its name because it was once believed that this system operated without our control. However, research has shown that a person can, in fact, control this part of the nervous system when provided with appropriate feedback of its functioning [70]. In pediatric gastroenterology, mind–body research suggests that biofeedback may be helpful in treating symptoms of IBS and functional abdominal pain (FAP) such as dyspepsia, bloating, and cramping [71,72].

#### Heart Rate Variability Biofeedback

Heart rate variability biofeedback (HRVB) is a type of biofeedback that can directly improve autonomic dysfunction and restore vagal tone [70]. Heart rate variability (HRV) is the change in time intervals between adjacent heartbeats. HRV can be measured non-invasively as a physiologic marker of autonomic nervous system function. Optimal HRV reflects a self-regulatory capacity, specifically a person’s adaptability and resilience to respond to and recover from stressful stimuli [73]. HRV varies from person to person depending on age, gender, health, and fitness. Lower HRV, or less variability in heart rate in various positions and activities, suggests higher persistent sympathetic tone and less resilience to stressful triggers [73]. Analysis of HRV involves power spectral analysis filtering techniques to separate the heart rate waveform into frequency ranges [74,75]. The high frequency (HF) band reflects parasympathetic activity, whereas the low frequency (LF) band reflects sympathetic activity [76]. The LF/HF ratio is used by some researchers to assess the balance between sympathetic and parasympathetic activity [74,77]. Non-invasive instruments that connect to an earlobe, finger, or to the chest via electrocardiogram leads measure HRV. The user then engages in breathing and relaxation exercises to visualize how this may affect their HRV directly on the screen [73]. HRVB is a novel modality and research on its efficacy and utilization is still emerging.

A study by Sowder et al. [72] investigated the effect of HRVB in children with FAP. Children participated in six sessions of HRVB aimed at normalizing autonomic balance. At baseline, children with FAP in this study had greater autonomic dysregulation than children without FAP, as indicated by a higher LF/HF ratio (*t*_(28)_ = 2.95; *p* = 0.006). After the treatment sessions, the FAP group was able to significantly reduce their symptoms which correlated with significant increases in their autonomic balance, as evidenced by a decrease in the LF/HF ratio(*t*_(19)_ = 2.57, *p* = 0.019). Another prospective open-label study by Stern et al. looked at 27 children with either IBS or FAP who underwent HRVB to treat their presenting symptoms for an average of eight 30-min sessions. They concluded that almost 70% of IBS patients and 63% of FAP patients achieved full remission (defined as having no symptoms for at least two full weeks) [71]. Qualitative data from this study showed that patients with both IBS and FAP expressed satisfaction with the intervention and their clinical outcomes and felt validated in the ‘reality’ of their symptoms.

Although no studies to date have looked at HRVB in IBD patients, a study by Jelenova et al. found that adolescents with mild IBD had less adaptability to stress compared to healthy controls [78]. The HRV of the IBD patients showed significantly lower levels of spectral activity in low frequency bands of HRV at both supine and standing positions (*p* < 0.005 and *p* < 0.01 respectively). This study was limited by the small sample size (25 IBD patients, 35 controls) and the fact that HRV was measured over five min, which may not be as accurate as 24 h measurements. Larger studies investigating normal values of HRV within healthy adolescent populations as compared to IBD populations would help to identify clinically significant HRV abnormalities within a pediatric IBD population. Although the effect of biofeedback on pediatric IBD has not yet been explored in depth, this modality offers a non-invasive way to decrease physiologic reactivity to stress, which may lead to IBD flares, decreased pain threshold, and IBS-IBD symptoms. Further research in this area is clearly warranted.

### 3.5. Yoga

Yoga is a mind–body technique arising from ancient India that combines physical postures, breath control, and meditation to promote health and relaxation. Yoga is believed to have mood-enhancing properties that inhibit physiologic stress and inflammation [79,80]. A systematic review of 25 randomized controlled trials provides evidence that yoga improves regulation of the sympathetic nervous system and hypothalamic–pituitary–adrenal system, as evidenced by multiple physiologic parameters including blood pressure, heart rate, cortisol, and cytokine expression [81]. Yoga also increases personal empowerment, which is the ability to develop the confidence and strength to set realistic goals and fulfill one’s potential [82]. Personal empowerment is a measure vital to one’s wellness and emotional health, and increased empowerment leads to improved patient activation. Of note, patient activation towards medical care can improve health outcomes, reduce medical system costs, and improve the patient’s overall healthcare experience [83,84]. Yoga is a low cost, high reward form of self-care that has the potential to fill a known health care gap by decreasing stress, promoting wellness, and reducing disease burden. 

Yoga protocols have been shown to be a feasible and safe adjunctive therapy for adolescents and young adults with IBS [85]. Nearly half of the participants in a brief yoga intervention for adolescents with IBS reported a significant reduction in pain and global symptom improvement, and young adults in the yoga group were significantly more likely to endorse symptom improvement compared to wait-list controls (*χ*^2^ = 11.13, *p* = 0.03) [85]. Another randomized trial of yoga for adolescents with IBS found significantly improved gastrointestinal symptoms (*p* < 0.01), lower levels of functional disability (*p* = 0.073), less use of emotion-focused avoidance (*p* = 0.09), and less anxiety (*p* = 0.09) compared to wait-list controls [86]. A systematic review of six randomized controlled trials studying yoga as a treatment for IBS in adults found significant improvements in quality of life, global improvement, and physical functioning [87]. However, the review was limited by the small overall sample size (total of 273 patients), heterogeneity of control groups, and unclear risk of bias in observed studies (no allocation concealment or adequate random sequence generation described). Despite these studies’ positive findings, there are no studies to date evaluating yoga as a potentially effective adjunct therapy for youth with IBD. Given the overlapping and interacting mechanisms between IBD and IBS, these preliminary yoga studies for IBS may also apply to the IBD population. Thus, there is a clear need for studies to investigate the potential benefits of yoga as a mind–body therapy in children and adolescents with IBD.

### 3.6. Clinical Hypnosis

Clinical hypnosis is a common MBI and refers to the specific ability to focus one’s attention narrowly and deepen one’s concentration, while simultaneously diminishing awareness of external stimuli. In this state of awareness, it is thought that the individual may be able to modulate some physiological processes not ordinarily under conscious control [88]. In the treatment of abdominal pain, most hypnosis or hypnotherapy studies have focused on the management of symptoms related to IBS, while a few have examined the action of hypnosis on IBD [89]. The strongest evidence for the efficacy of hypnotherapy in IBD is its association with reduced IBD related inflammation and improved quality of life. Mixed results have been obtained regarding its effects on psychological and pain outcomes [90]. The mechanism of action of hypnotherapy is believed to be via the ‘brain–gut axis’, through the modulation of vagal visceral afferent signals via the pontomedullary nuclei, limbic regions including the amygdala, hippocampus, and insula, and vagal efferent signaling via pathways of descending modulation. These pathways may be dysregulated by cognitive and environmental demands, leading to the overriding of local reflex functions that occur during sleep or in the context of digestion [11].

Hypnotherapy normally begins with an introduction by the practitioner, development of rapport, and receipt of permission to perform the hypnosis exercise. Following, a hypnosis induction is performed, with the aim of allowing the individual to enter an altered consciousness or trance state (e.g., similar to a narrow state of focused attention). This state is readily achieved by children, who naturally are able to enter a state of focus and attention during play or while engaging their imagination. Once in the hypnotic state, gentle suggestions are made for enhancing one’s ability to regulate or normalize gastro-intestinal function, including the minimization of pain, nausea, vomiting or other symptomatology. Various metaphors can be employed to facilitate this process; for example, the gut may be likened to a smoothly flowing river, whose flow can be guided by the patient’s gentle encouragement. The placing of the patient’s hands over the abdomen, or taking specific medicines, can be seen as a way to engender calm and protection against pain, bloating, discomfort, or abnormal bowel habits. Subsequently, patients are encouraged to utilize the technique to calm their symptoms at other times during their daily life. Hypnotherapy has been demonstrated to be safe and effective for use in youth; however, it should only be employed by appropriately trained individuals [91].

In a randomized controlled study of 54 adult patients with ulcerative colitis, clinical remission was prolonged in 26 patients by a mean of 78 days compared to 29 attention controls. The intervention consisted of a seven-session standardized treatment protocol. Patients were also provided with an audio recording to practice outside of sessions five days per week during the study. The control intervention consisted of non-directive discussion about Ulcerative Colitis and the ‘mind–body connection’. In addition, analysis of the group maintaining remission at one year showed a significantly higher remission rate among those in the hypnosis condition (68%) compared to control patients (40%) [92]. While mechanisms of action are unknown, stress management and increased self-efficacy have been posited as possible mediators.

In another study, 15 adult patients with severe or very severe IBD on corticosteroids, but not responding to medication, received 12 sessions of ‘gut-focused hypnotherapy’ and were followed up for a mean duration of 5.4 years. Sixty percent of patients were able to entirely stop corticosteroids and did not require them at follow-up [93]. In a similar study, 15 patients with severe IBD underwent 12 sessions of gut directed hypnosis, which focused on tactile stimuli as well as visualization. In 14 out of the 15 patients, disease severity was reduced from severe to either mild or moderate following treatment, and quality of life was improved from ‘very poor’ and ‘poor’ to ‘good’ and ‘excellent’ [94]. Of note, one session of ‘gut-directed hypnotherapy’ has been shown to reduce systemic and rectal mucosal inflammatory responses. Serum interleukin-6 concentration was reduced by 53%, circulating natural killer cell numbers were reduced by 18%, rectal mucosal release of substance P was reduced by 81%, histamine was reduced by 35%, and interleukin-13 was reduced by 53% [95].

In the pediatric population, hypnotherapy was successfully utilized in a case series for six children with IBD. The children received between four and 12 sessions over three months, and treatment resulted in improved symptoms and decreased inflammatory markers, though the exact values are not provided in the report [96]. In a randomized controlled study of 53 pediatric patients (aged eight to 18) with IBS and functional abdominal pain, patients underwent six sessions of hypnotherapy (HT) over a three-month period. The control intervention was standard medical care, with six sessions of supportive therapy. Pain intensity decreased in the HT group from 13.5 to 1.3 and in the control group from 14.1 to 8 (*p* < 0.001). Pain frequency reporting also decreased in the HT group from 13.1 to 1.1 and in the control group from 14.4 to 9.3 (*p* = 0.007). The three-month pain remission rate was 59% in the HT group compared to 12% in the standard care group (*p* < 0.001). Interestingly, there continued to be post-treatment improvement and remission rates at one year (85% and 25% respectively; *p* < 0.001) [97].

While no larger studies have investigated how some of these effects might be mediated in children with IBD, an interesting study in adults with IBS showed that gut-directed hypnotherapy had a normalizing effect on the aberrant central processing of visceral signals. Forty-four female adult patients were randomized to hypnosis or educational therapy. They received seven weekly one hour long sessions of hypnotherapy, including CD recordings for home use. Rectal distention was performed while the patient underwent functional magnetic resonance imaging of the brain to assess the central nervous system response to the distention stimulus. Both groups showed reduction in blood oxygen level dependent signals, including anterior insula, albeit by different centrally acting mechanisms [98]. The normalization of autonomic nervous system functions may also play a role in symptom management, as gut directed hypnotherapy has been shown to reduce heart and respiratory rates independently of emotional content [99]. Hypnotherapy is a promising therapy that may support symptom management in inflammatory bowel disease and potentially decrease the inflammatory response itself. More studies are needed to explore the feasibility, efficacy, and dose-response relationship of hypnotherapy interventions among pediatric patients suffering from IBD.

## 4. Sample Case

A 14-year-old female with a diagnosis of ileocolonic Crohn’s disease for one year presented for a second opinion for IBD evaluation and management. The important findings on initial endoscopy were chronic ileitis of the terminal ileum, moderately active chronic colitis of the ascending colon, and colonic mucosa with rare branching crypt. Magnetic resonance enterography demonstrated a segment of borderline mild circumferential thickening of the small bowel in the ileum. Previous laboratories showed an elevated C-reactive protein (17 mg/L) and erythrocyte sedimentation rate (42 mm/h), fecal calprotectin (839 mcg/g), and low vitamin D (12 ng/mL). Her weight was 48 kg (24%), and her BMI was 21.28 kg/m^2^ (50%). She was initially started on sulfasalazine, folic acid, and prednisone taper with the plan to transition to 6-mercaptopurine. However, she was only currently compliant with iron supplementation due to concerns about medication side effects.

The patient’s presenting symptoms included watery diarrhea two times daily without hematochezia, frequent abdominal pain limiting her activity, and fatigue requiring a three hour nap every afternoon. She described significant stress related to Crohn’s and school and became tearful when discussing her disease and desire to be normal. She also endorsed medical-related anxiety, needle phobia, and increasing frustration about her recurrent abdominal pain. During the evaluation, disease management options were discussed, including biologics, re-induction with steroids, use of immunomodulators, and enteral therapy. The patient was also referred for psychotherapy with a pediatric pain psychologist.

Psychological evaluation suggested significant academic and social anxiety related to IBD. Specifically, the patient endorsed difficulties concentrating in class due to symptoms, embarrassment about bathroom needs at school, body image concerns around peers, pain catastrophizing when out with friends, and anxiety around upcoming required blood draws. Weekly psychotherapy was initiated, and treatment goals included teaching her anxiety and pain management strategies, increasing daily functioning, and supporting her adjustment to life with a chronic illness.

Treatment initially focused on validating her past experiences, supporting increased trust with medical professionals, and empowering her to be more engaged in her health care. The mind–body modalities used in treatment included: (1) biofeedback for stress and pain management (e.g., HRV via emWave—a commonly used biofeedback program by HeartMath LLC, Boulder Creek, CA, USA); (2) hypnosis for needle phobia and pain management (e.g., comfort and relaxation dials in her ‘control center’, the brain); (3) mindfulness for stress and anxiety management; (4) CBT for needle phobia (e.g., graded exposures to feared stimulus like needles and blood draw); (5) and CBT for general anxiety and pain management (e.g., increasing awareness of negative pain-related thoughts, cognitive restructuring, behavioral activation to increase daily functioning).

During the first month of treatment, each session began with biofeedback and/or hypnosis exercises, such as playing the Garden Game on emWave (HeartMath LLC, Boulder Creek, CA, USA) or utilizing her relaxation dial to release worries and tension. CBT techniques were incorporated into the last 30 min of the session, focusing on graded needle exposures. For example, she practiced looking at pictures of blood draws and preparing her arm for a blood draw, with rubbing alcohol and tourniquet placement, and applying relaxation-based coping skills. Daily homework included practicing mind–body skills using biofeedback applications such as Bellybio [100] and Breathe2Relax [101], listening to a hypnosis recording about her comfort dial, and creating a weekly behavioral activation log. Treatment focused on a top-down and bottom-up approach to anxiety and pain management. After four weekly sessions working with these mind–body interventions, the patient was able to have her blood drawn with minimal difficulty. She also endorsed less anxiety at school and spent more time out of the house with friends.

At the patient’s follow-up gastroenterology visit, she agreed to restart disease modifying medications. Of note, her calprotectin values subsequently normalized to 70 mcg/g, and her anemia and inflammatory markers also improved. The patient continued psychotherapy for 12 weeks. This multidisciplinary approach allowed for shared decision making in a case where the patient and family had at first demonstrated medical noncompliance due to medical-related anxiety. This case highlights the benefit of integrating mind–body interventions into IBD treatment.

When considering this case, and the biopsychosocial challenges associated with IBD, it is also important to highlight that ongoing psychotherapy is often recommended by multidisciplinary IBD teams to support mood, IBD symptom management, and health promotion (e.g., medication adherence, transitioning care). As studies have demonstrated, psychotherapy can have long-term positive effects on psychological and IBD-related outcomes. While most empirical evidence supports CBT for youth with IBD, acceptance and mindfulness-based treatment strategies should be considered when working with patients on adjustment and identity concerns related to IBD. Continued research in this area is warranted.

## 5. Conclusions

Mind–body interventions may be an effective non-invasive adjunctive therapy to standard medical care in pediatric IBD populations. Research shows that psychotherapy, specifically cognitive behavioral therapy, may be helpful in improving depression, functioning, and quality of life in youth with IBD. Relaxation and mindfulness-based interventions appear to improve psychological functioning among adult IBD populations, with particular benefit for subpopulations of patients experiencing heightened states of stress and concurrent IBS symptoms. Heart rate variability biofeedback and yoga may offer a way to help patients decrease pain and IBS-IBD symptoms, as well as improve anxiety and elevated persistent sympathetic tone. Preliminary data also suggests that clinical hypnosis and other mind–body interventions may have the potential to affect IBD activity (e.g., reduced inflammatory response). Further research needs to be conducted in pediatric samples in order to further validate the feasibility, efficacy, and cost effectiveness of these mind–body interventions in pediatric IBD. Manipulative therapies, such as acupuncture and massage, and dietary therapies may also improve overall well-being in IBD patients and be an area worthy of further investigation.

Mind–body interventions can be implemented in the clinical setting by various providers, including GI physicians, nurse practitioners, and physician assistants. Mental health providers such as social workers, pediatric pain psychologists, or clinical psychologists can also be trained in mind–body interventions. For the time being, insurance may not cover specific mind–body treatments. However, providers often can bill for services through evaluation and management codes based on time (for medical providers) or health and behavior codes based on time (for mental health providers). As evidence emerges on the efficacy of mind–body interventions to improve IBD outcomes, hopefully insurance companies will deem these interventions as medically necessary and gradually incorporate them into part of mainstream care.

We encourage pediatric IBD providers to reflect on the importance of the brain–gut connection with patients and families, assess their interest in mind–body interventions, and engage in multidisciplinary treatment with mind–body treatments when appropriate and supported by empirical evidence. This integrative treatment approach has the promise to improve patients’ disease activity and overall well-being.
